# A chromosome-level genome assembly of *Stenchaetothrips biformis* and comparative genomic analysis highlights distinct host adaptations among thrips

**DOI:** 10.1038/s42003-023-05187-1

**Published:** 2023-08-04

**Authors:** Qing-Ling Hu, Zhuang-Xin Ye, Ji-Chong Zhuo, Jun-Min Li, Chuan-Xi Zhang

**Affiliations:** 1 State Key Laboratory for Managing Biotic and Chemical Threats to the Quality and Safety of Agro-Products, Key Laboratory of Biotechnology in Plant Protection of Ministry of Agriculture and Zhejiang Province, Institute of Plant Virology, Ningbo University, Ningbo, 315211 China; 2 Institute of Insect Science, Zhejiang University, Hangzhou, 310058 China

**Keywords:** Comparative genomics, Evolutionary genetics

## Abstract

Insects have a limited host range due to genomic adaptation. Thysanoptera, commonly known as thrips, occupies distinct feeding habitats, but there is a lack of comparative genomic analyses and limited genomic resources available. In this study, the chromosome-level genome of *Stenchaetothrips biformis*, an oligophagous pest of rice, is assembled using multiple sequencing technologies, including PacBio, Illumina short-reads, and Hi-C technology. A 338.86 Mb genome is obtained, consisting of 1269 contigs with a contig N50 size of 381 kb and a scaffold N50 size of 18.21 Mb. Thereafter, 17,167 protein-coding genes and 36.25% repetitive elements are annotated. Comparative genomic analyses with two other polyphagous thrips, revealing contracted chemosensory-related and expanded stress response and detoxification gene families in *S. biformis*, potentially facilitating rice adaptation. In the polyphagous thrips species *Frankliniella occidentalis* and *Thrips palmi*, expanded gene families are enriched in metabolism of aromatic and anthocyanin-containing compounds, immunity against viruses, and detoxification enzymes. These expansion gene families play crucial roles not only in adapting to hosts but also in development of pesticide resistance, as evidenced by transcriptome results after insecticides treatment. This study provides a chromosome-level genome assembly and lays the foundation for further studies on thrips evolution and pest management.

## Abstract (web summary)

A chromosome-level genome assembly of the rice crop pest, *Stenchaetothrips biformis*, and comparative genomic analysis to two other thrip species highlights adaptive differences among these thrips and may inform future pest control strategies.

## Introduction

The order Thysanoptera, commonly referred to as thrips, comprises over 7000 species, with a body length ranging from 1 to 3 mm^1,2^. Thrips exhibit diverse biological features, with approximately half of the known species feeding on fungi and a few feeding on small arthropods^3^. The remaining species are phytophagous, with some capable of causing harm to agricultural and horticultural crops. Examples of such species include *Frankliniella occidentalis*, *Thrips palmi*, *Stenchaetothrips biformis*, and *Thrips tabaci*. Despite their ecological importance, there is a dearth of genomic resources to facilitate a better understanding of genetic and molecular mechanisms underlying thrip’s adaptation to their different hosts.

**Fig. 1:**
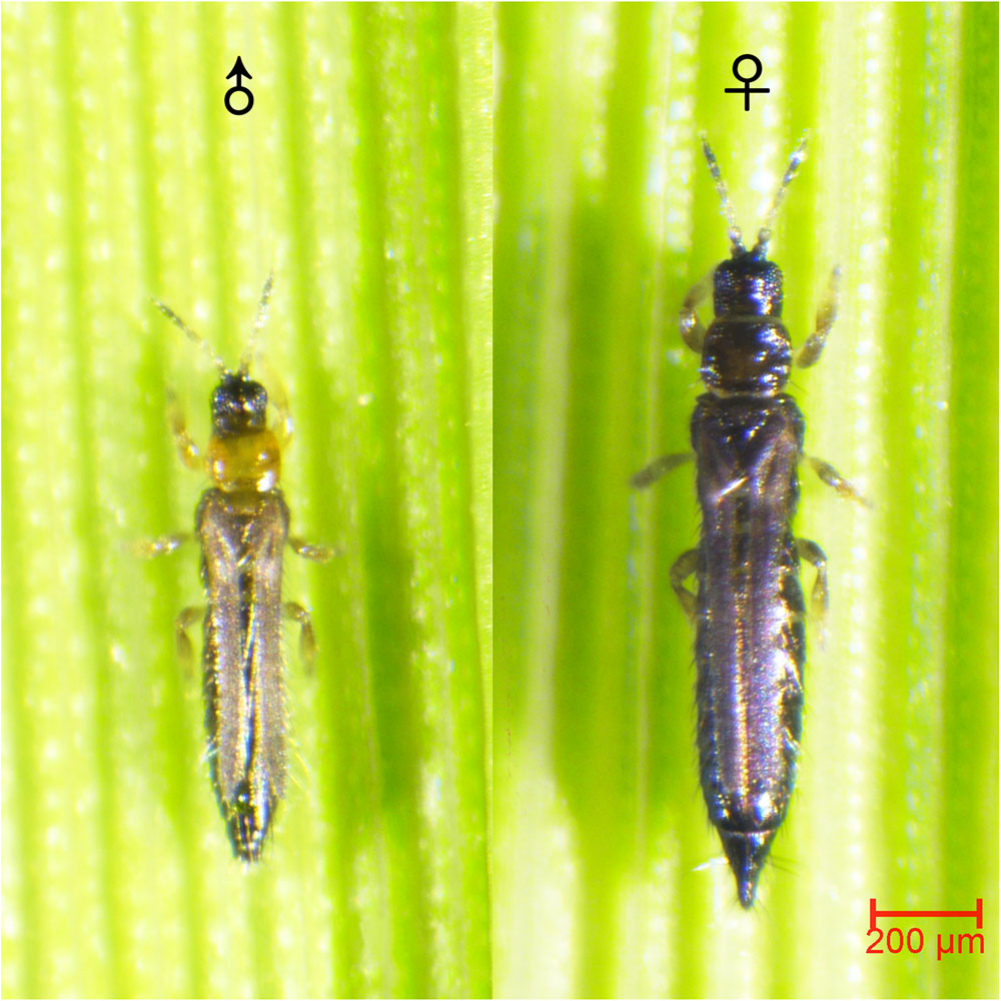
Image of male and female adult *S. biformis* taken by the color stereoscope microscope. Scale bar: 200 μm.

*S. biformis* (Thysanoptera: Thripidae), commonly known as rice thrips, is a highly destructive pest to rice crops (Fig. 1). The species has a wide distribution in Asia, Europe, Oceania, and South America, as documented in several studies^4–6^. Although *S. biformis* is oligophagous and can infest various Poaceae species, including wheat, barley, sugarcane^4^, and *Leersia hexandra*, it is infamous for its severe damage to rice. The pest uses its ‘punch and suck’ mouthparts to primarily attack the young leaves of rice during the seedling and tillering stages. The attack can result in leaf rolling, discoloration, and even whole plant wilting. Since the 1970s, *S. biformis* has been responsible for yield losses in rice production in several Asian countries. Currently, the genome of *S. biformis* has not been reported yet.

*S. biformis* exhibits biological differences when compared to the other two thrips species, *F. occidentalis* and *T. palmi*, whose genomes have been reported. These variations include host range, feeding habits, and the viruses and symbiotic bacteria they carry. Specifically, *S. biformis* predominantly feeds on young rice leaves, whereas *F. occidentalis* feeds on a wider range of crops, including vegetables and ornamental plants^7^. *T. palmi*, on the other hand, has a relatively smaller host range and mainly causes damage to the fruits, leaves, and flowers of vegetables, as well as a few ornamental plants such as orchids^8^.

*S. biformis* has not been reported as a vector for any virus, unlike *F. occidentalis*, which is known to transmit at least eleven plant viruses^9^, including the *tomato spotted wilt orthotospovirus* (TSWV)^10^. Additionally, *T. palmi* is a vector for transmitting several plant tospoviruses, including the *groundnut bud necrosis virus* (GBNV)^11^ and *watermelon bud necrosis virus* (WBNV)^12^, both of which can cause severe crop yield losses. Furthermore, two types of bacterial symbionts belonging to the family, Enterobacteriaceae, have been found in the hindgut and *Malpighian tubeles* of *F. occidentalis*^13^. These symbionts are advantageous for *F. occidentalis* under conditions of food scarcity^14^. In contrast, up to date, there are no reports of bacterial symbionts in either *T. palmi* or *S. biformis*.

Genomic adaptive evolution to host plants is a common phenomenon in nature for insect herbivores. The molecular interaction mechanisms between insects and plants typically involve the perception of host plants, the response of salivary proteins to plant defenses, digestion of plant tissues, and detoxification of plant secondary metabolites^15^. These mechanisms can lead to the specialization and speciation of insect herbivores. Although the genome of *F. occidentalis*^16^ and *T. palmi*^17^ have been established, no comparative genomic analysis has yet revealed the underlying genetic mechanisms of different features among the Thripidae species.

This study presents the genome of *S. biformis*, which was de novo assembled using a combination of PacBio and Illumina sequencing technologies and further assembled at the chromosome level by means of Hi-C technology. Moreover, genome annotation and comparative genomic analysis were conducted, with a focus on the genetic basis of different biological attributes observed in *S. biformis*, *T. palmi*, and *F. occidentalis*. The findings of our study provide a valuable genomic resource for understanding the genetic, evolutionary, and ecological issues of thrips and further offer a possibility to implement integrated pest management of these pests.

## Results

### Genome sequencing and assembly

After filtering the adapters and low-quality reads, approximately 30.58 Gb of clean reads (~100×) were retained by Illumina paired-end short-reads sequencing, and about 63.24 Gb of subreads (~200×) were obtained by PacBio long-reads sequencing. The PacBio reads had an average length of 16.34 kb, with an N50 length of 19.57 kb (Supplementary Data 1).

Using gce v1.0.2, the *S. biformis* genome was estimated to be approximately 251.50 Mb, with a heterozygosity rate of 0.63% and repeat content of 31.5%, based on Illumina short reads when kmer was set to 17 (Supplementary Data 2; Supplementary Fig. 1). The PacBio subreads were then primarily assembled and polished to obtain the raw contigs. Typically, the raw-contig genome was about 751.60 Mb, which contained 6040 contigs with a contig N50 length of 205.12 kb. After removing haploids, a haploid-contig level genome assembly with a length of 337.63 Mb was obtained, consisting of 1267 contigs with a contig N50 length of 381.39 kb (Supplementary Data 3).

Upon sequencing the Hi–C library, a total of 75.16 Gb paired-end reads were generated (Supplementary Data 1). Subsequently, the Hi–C data were utilized to enhance the genome at the chromosome level. The final genome was 338.86 Mb in length, which consisted of 31 scaffolds with a scaffold N50 of 18.21 Mb (Table 1). A total of 18 chromosome-level scaffolds (length range, 14.51–27.23 Mb) were assembled (Supplementary Fig. 2), accounting for 99.8% of the whole genome. Additionally, there were 13 small scaffolds, taking up ~0.2% of the genome. The final genome assembly of *S. biformis* was larger than the estimated size, likely due to the impact of heterozygous regions assembling into different genomic regions and forming duplications, thereby affecting the accuracy of genome estimates. The genome GC content of *S. biformis* was 51.09%.

**Table 1:** Genome assembly and annotation statistics of *S. biformis*.

Statistic	Value
*Genome assembly*	
Base pairs (Mb)	338,862,303
% Ns	0.36
Contig numbers	1,269
contigN50 (kb)	381,394
Scaffolds numbers	31
Scaffold N50 (kb)	18,206,519
GC (%)	51.09
% of scaffolds anchored in chromosomes	99.81
BUSCO completeness (%)	96.60
*Gene annotation*	
Protein-coding genes	17,167
Mean protein length (aa)	574.70
Mean gene length (bp)	7,359.18
Exons per gene	7.33
Exon (%)	8.71
Mean exon length (bp)	235.65
BUSCO completeness (%)	92.80

To evaluate the completeness of the *S. biformis* genome assembly, BUSCO completeness analysis was performed using eukaryota, arthropoda, and insecta databases. The analysis showed that the genome completeness in the insecta database was 96.6%, including 91.3% single-copy and 5.3% duplicated genes. In comparison, only 1% and 2.4% of genes were fragmented and missing, respectively (Table 1, Supplementary Fig. 3a).

### Gene annotation

**Fig. 2:**
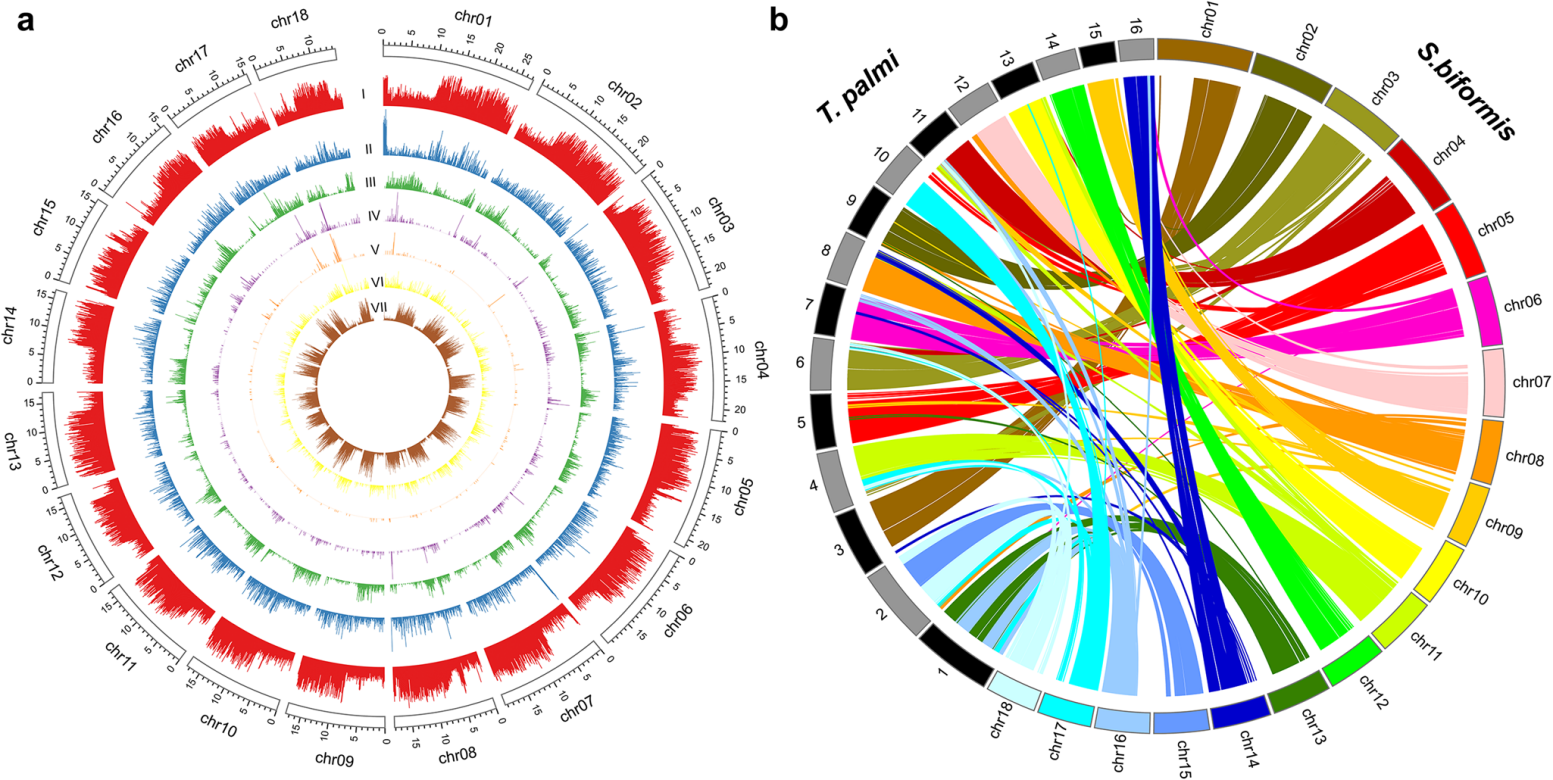
Chromosome-level genome assembly of *S. biformis*. **a** Ideograms of 18 chromosomes of *S. biformis* show with genomic features. I, GC content across the genome; II, protein coding genes counts; III, density of repeat contents DNA transposons; IV, density of repeat contents LINEs, long interspersed elements; V, density of repeat contents SINEs, short interspersed elements; VI, density of repeat contents LTR, long terminal repeat elements; VII, density of simple repeats. **b** Chromosome-scale synteny blocks between *S. biformis* and *T. palmi*.

In total, 36.25% of repetitive elements were identified from the genome of *S. biformis*, including 0.07% of short interspersed nuclear elements (SINEs), 1.02% of long interspersed nuclear elements (LINEs), 3.18% of long terminal repeats elements (LTRs), 4.30% of DNA transposons, and 23.41% of unclassified elements. In addition, 1907 Satellites and 230,843 Simple Repeats were identified, accounting for 0.19% and 3.45% of the genome, respectively (Fig. 2a, Supplementary Data 4).

To improve the accuracy of gene annotation, Illumina RNA-seq data from male and female libraries were sequenced separately. After quality control, ~14.26 Gb of clean data were kept, comprising 96,169,734 sequences with an average read length of 148 bp (Supplementary Data 1). These short reads were subsequently assembled into 170,763 transcripts using Trinity for genome annotation.

A total of 17,176 protein-coding genes were annotated in the genome of *S. biformis* (Table 1, Supplementary Data 5). The average gene length and exon length were 7359 bp and 235 bp, respectively. The average exon number per gene was 7.33 (Supplementary Data 5). Based on BUSCO analysis, the completeness of protein-coding genes was evaluated to be 92.8% by aligning against the insecta_odb10 database (Supplementary Fig. 3b). Functional annotation demonstrated that 15,707 (91%), 15,730 (92%), 14,317 (83%), and 11,107 (65%) genes were significantly aligned in the NR, UniprotKB/TrEMBL, UniprotKB/Swiss-Prot, Interproscan, and eggnog databases, respectively (Supplementary Data 6). There were 7958 (46%) and 8354 (49%) genes annotated to KEGG pathways and GO terms, respectively. In total, 16,195 (94%) genes occurred at least once in all the databases (Supplementary Data 6). The high completeness rate of BUSCO and functional annotation rate of the gene set indicated the accuracy and reliability of our genome annotation results. Additionally, non-coding RNAs (ncRNAs) were identified across the genome. In total, 91 microRNAs (miRNAs), 660 ribosomal RNAs (rRNAs), and 188 small nuclear RNAs (snRNAs) were identified based on the Rfam databases, while 2572 transfer RNAs (tRNAs) were identified using tRNAscan-SE database (Supplementary Data 7).

### Synteny, orthology, and phylogenetic relationships

Comparative genomics analyses were carried out on *S. biformis* and 18 other insect species representing various orders, including Hymenoptera, Neuroptera, Lepidoptera, Hemiptera, Coleoptera, Diptera, Ephemeroptera, Phthiraptera, and Siphonaptera (Supplementary Data 8). As a result, OrthoFinder revealed that 276,424 (89.2% of all) genes from 18 species could be clustered into 22,300 orthogroups (Supplementary Data 9). Out of these orthogroups, 2432 were present in all species, and 298 were single-copy orthogroups (Supplementary Data 9). For *S. biformis*, 16,499 out of 17,167 genes were assigned to 9646 orthogroups, 331 of which were species-specific and contained 1168 genes in total (Supplementary Data 10).

**Fig. 3:**
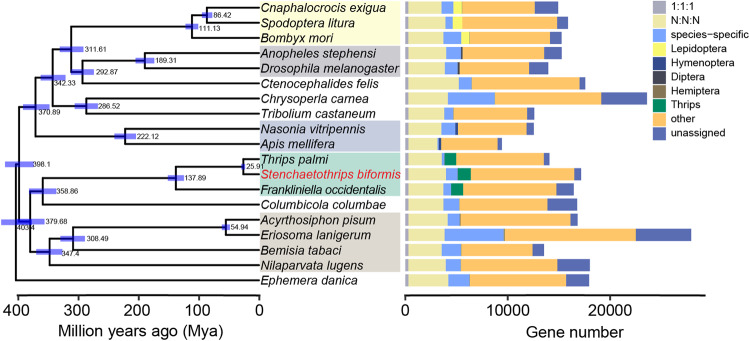
Phylogenetic relationships and gene orthology among thrips and other insect species. The approximately-maximum-likelihood phylogenetic tree was constructed based on 298 single-copy orthologous genes among 19 species. The divergence time and the 95% confidence interval (blue bar) were shown at the internodes. Bars represent the gene number of different types of orthologues. 1:1:1 (single copy orthologous genes in all species); N:N:N (multi-copy orthologous genes in all species); species-specific (unique genes to specific species); Lepidoptera (Lepidopteran-specific orthologous genes); Hymenoptera (Hymenopteran-specific orthologous genes); Diptera (Dipteran-specific orthologous genes); Hemiptera (Hemipteran-specific orthologous genes); Thrips (Thrips-specific orthologous genes); other (genes belong to all other orthogroups); unassigned (genes cannot be assigned to any orthogroups).

A species phylogenetic tree was also constructed based on 298 single-copy genes, which suggested that Thysanoptera was the sister group of Phthiraptera (Fig. 3). The inferred divergence time of Thysanoptera differentiating from Phthiraptera was about 358.86 Ma, while that from Hemiptera was around 379.68 Ma (Fig. 3), consistent with previous studies^18,19^. The study also sheds light on the speciation times within Thysanoptera species. *F. occidentalis* diverged from the other two species around 137.89 Ma, and *T. palmi* split with *S.biformis* at ~25.91 Ma (Fig. 3). The phylogenetic relationship was consistent with the previous one based on five molecular genetic loci^2^.

The chromosome-level genome assembly of *S. biformis* was compared with that of *T. palmi*, and good collinearity was found between them (Fig. 2b). However, 18 chromosome-level scaffolds were constructed in *S. biformis* compared with 16 chromosomes in *T. palmi*. Besides, the chr13 and chr16 in *S. biformis* showed syntenic blocks with chromosome 1 in *T. palmi*, while chr15 and chr18 in *S. biformis* were collinear with chromosome 2 in *T. palmi*. More investigations are warranted to analyze the underlying evolutionary importance.

### Gene family expansion and contraction

**Fig. 4:**
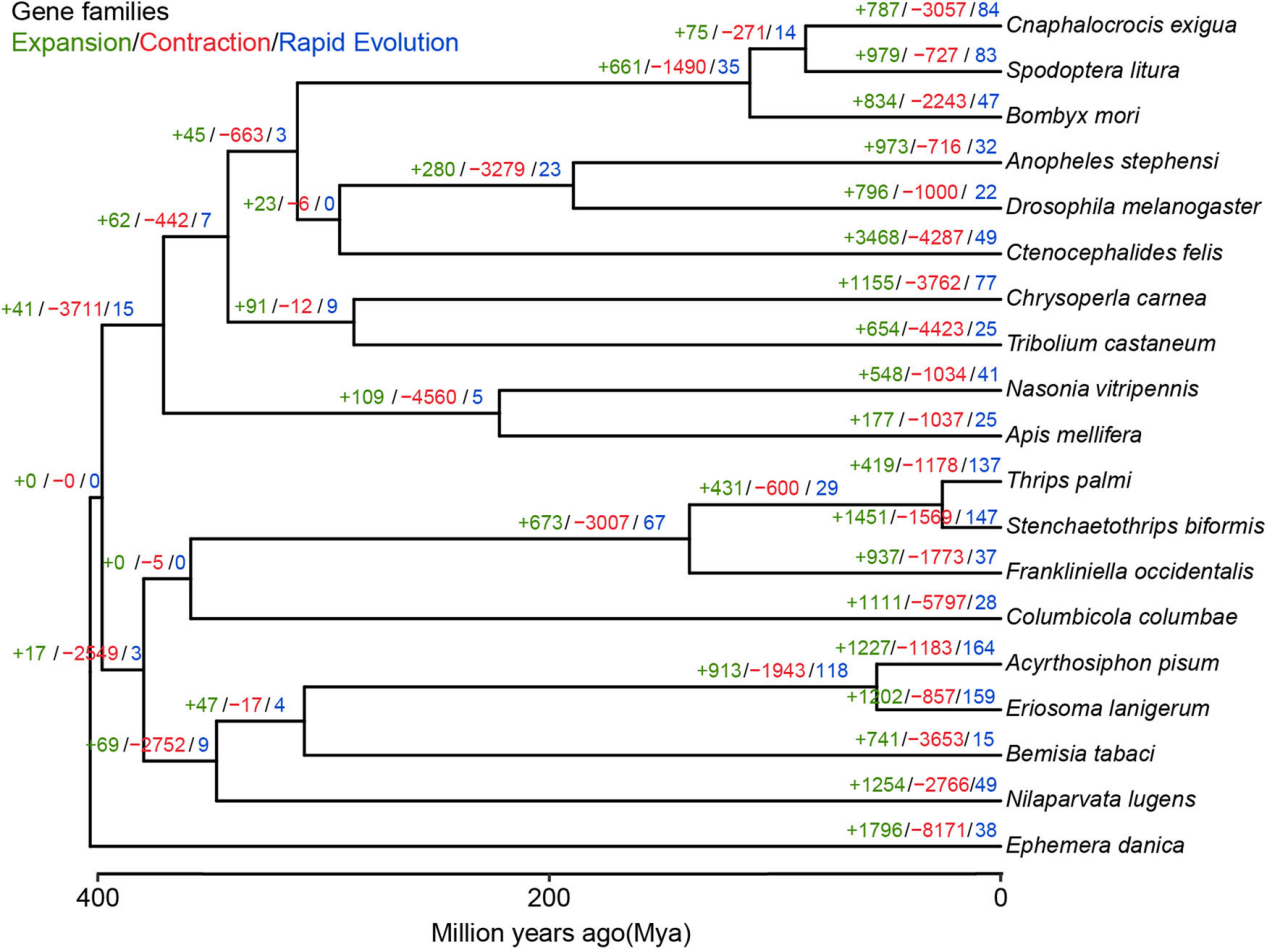
Gene family evolution among *S. biformis* and 18 other insect species. The numbers within each node were extracted using CAFE software, with green indicating the orthogroups expansions, red representing the orthogroups contractions, and blue indicating the orthogroups rapid evolutions.

CAFE v4.2.1 was employed to analyze the expansion and contraction of gene families (orthogroups) characterized by OrthoFinder. Our results revealed that *S. biformis* had 1451 expanded and 1569 contracted gene families compared to the common ancestor of *T. palmi* and *S. biformis* (Fig. 4), and among them, 126 orthogroups were significantly expanded (*p*-value < 0.01). GO analyses indicated that the expanded orthogroups were significantly enriched (*q*-value < 0.05) in biological processes, such as chromatin modification (GO:0016568) and epigenetic regulation (GO:0040029), metabolic processes (lipid metabolism (GO:0006629) and carbohydrate derivative catabolism (GO:1901136)), and stress responses (response to ethanol (GO:0045471), vitamin (GO:0033273), hypoxia (GO:0001666), cocaine (GO:0042220), antibiotic (GO:0046677)) (Supplementary Data 11, Fig. 5). Furthermore, KEGG pathway analysis showed that the expanded orthogroups were enriched in immune system functions (neutrophil extracellular trap formation (ko04613) and antigen processing and presentation (ko04612)), amino acid metabolism (cysteine and methionine metabolism (ko00270)), carbohydrate metabolism (fructose, mannose (ko00051), and galactose (ko00052) metabolism), digestive system functions (cholesterol (ko04979), carbohydrate (ko04973), vitamin (ko04977), and mineral digestion and absorption (ko04978)), and environmental adaptation (thermogenesis (ko04714)) (Supplementary Data 12). These results suggest that the expanded gene families in *S. biformis* were associated with the digestion and metabolism of food and the adaptation to the environment.

**Fig. 5:**
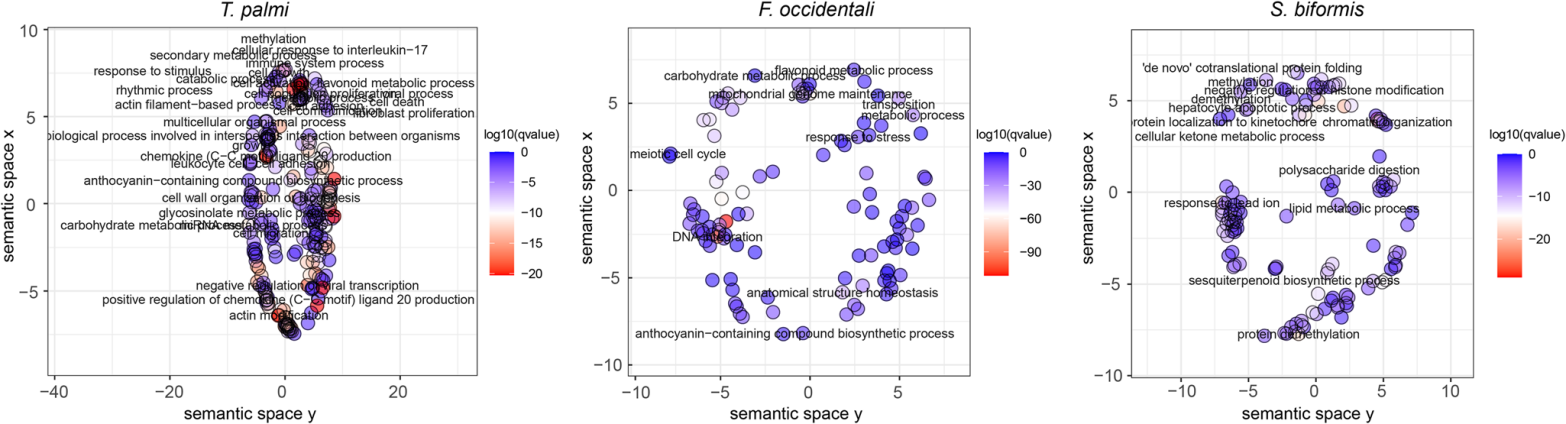
GO terms for significantly expanded orthogroups in three thrips species. GO significantly enriched terms (*q*-value < 0.05) were displayed using REVIGO^80^ to remove the redundant terms. The text-labeled terms were with dispensability (the semantic similarity threshold) less than 0.1.

In the genome of *T. palmi*, 419 gene families were expanded while 1178 were contracted (Fig. 4). Among these, 32 gene families were significantly expanded, and 105 were significantly contracted (*p*-value < 0.01). Similarly, *F. occidentalis* had 34 significantly expanded and 3 significantly contracted orthogroups (Fig. 4). In contrast to *S. biformis*, which primarily feeds on a limited number of Poaceae species, both *T. palmi* and *F. occidentalis* feed on a wide range of vegetables and flowers, which contain the water-soluble pigment anthocyanin, giving them their colorful appearance. Notably, the expanded gene families in both *T. palmi* and *F. occidentalis* were enriched in metabolic processes related to aromatic (GO:0019439, GO:0006725) and anthocyanin-containing compounds (GO:0046283), which could be associated with the metabolism of anthocyanin from the host plant, as suggested by GO analyses (Supplementary Data 13 and 15). Additionally, KEGG analyses revealed that the expanded gene families were significantly enriched (*q*-value < 0.05) in lipid metabolism (fatty acid degradation (ko00071)), carbohydrate metabolism (galactose metabolism (ko00052), amino sugar and nucleotide sugar metabolism (ko00520)), and amino acid metabolism (lysine degradation (ko00310), glutathione metabolism (ko00480), cyanoamino acid metabolism (ko00460)), which could be related to host plant metabolism (Supplementary Data 14).

Furthermore, *T. palmi* and *F. occidentalis* were both vectors of different types of destructive plant viruses. Thrips transmit tospoviruses in a persistent-propagative way, by which viruses replicate in the midgut and salivary glands of thrips. The immune system of *F. occidentalis* and *T. palmi* was also proven to be activated after virus infection. Medeiros et al. ^20^. analyzed the transcriptome of *F. occidentalis* after TSWV infection and found that the upregulated genes were involved in antimicrobial peptide encoding, pathogen recognition, and innate immune response. In this study, we found expanded gene families were enriched in viral transcription and immune response, such as T cell activation (GO:0002286), B cell activation (GO:0042113), regulation of apoptotic (GO:0042981), and mitophagy (GO:0000422) in *T. palmi* (Supplementary Data 13). KEGG pathways enriched in the immune system of NOD-like receptor signaling pathway (ko04621) and complement and coagulation cascades (ko04610) (Supplementary Data 14). In *F. occidentalis*, gene families were enriched in innate immune response (GO:0045087) and antibacterial peptide production (GO:0002778) (Supplementary Data 15), and KEGG pathways were enriched in Toll and Imd signaling pathway (ko04624) (Supplementary Data 16). The expansion of immune-related genes in *T. palmi* and *F. occidentalis* may be involved in the transmission of viruses. Bacterial symbionts reside in the hindgut and malpighian tubules of *F. occidentalis*, and expanded gene families were found to be enriched in the peptidoglycan catabolic process (GO:0009253) (Supplementary Data 15), which may aid in the coexistence of *F. occidentalis* with gut bacteria.

*F. occidentalis* and *T. palmi* are both polyphagous pests, which require a more potent detoxification enzyme system than oligophagous pests to adapt to the wider range of host plants. Insects use metabolic adaptation mechanisms such as P450s and glutathione-S-transferase to detoxify the plant’s secondary metabolites and improve insecticide resistance. In this study, the KEGG pathways of xenobiotics biodegradation and metabolism, including drug metabolism-cytochrome P450 (ko00982), drug metabolism-other enzymes (ko00983), and biosynthesis of other secondary metabolites (ko00999), were found to be significantly enriched in *F. occidentalis* and *T. palmi* (Supplementary Data 14 and 16). The results suggest that gene family expansion may be related to genome adaptation to host plant digestion, response to virus and bacterial infection, and metabolism of plant secondary metabolites in *F. occidentalis* and *T. palmi*. However, further research is necessary to elucidate the specific molecular mechanisms of these genes and provide a basis for managing pest and virus transmission.

### Chemosensory-related genes/detoxification-related genes

**Fig. 6:**
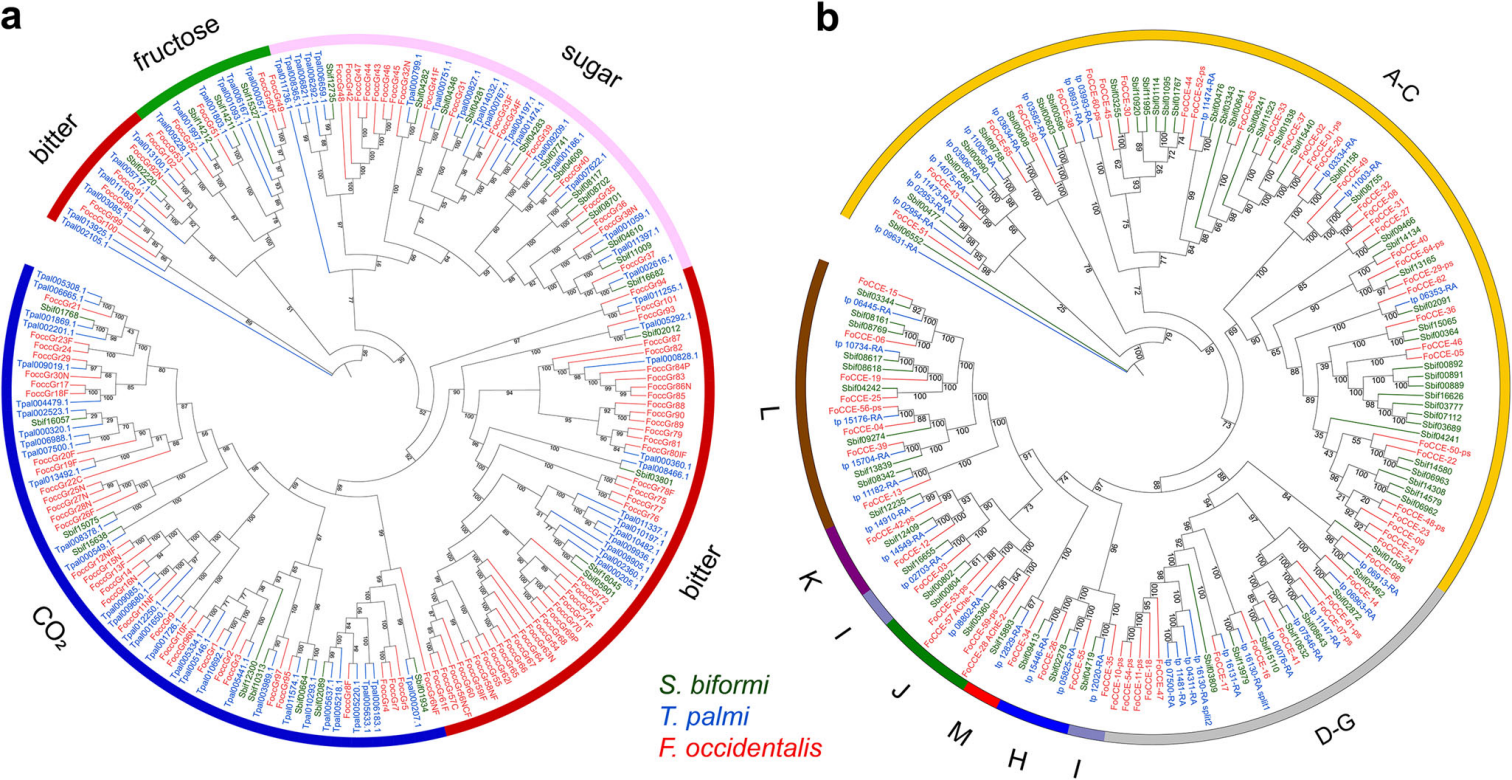
Phylogenetic tree of GRs and CCEs. Maximum-likelihood (ML) phylogenetic trees of **a** GRs and **b** CCEs genes annotated in three thrips were constructed using IQ-TREE with 1000 bootstrap replicates.

Given that host adaptation usually involves host recognition and detoxification of host secondary metabolites, this study manually annotated the common gene families, including chemosensory-related genes of gustatory receptors (GRs), odorant receptors (ORs), ionotropic receptors (IRs), chemosensory proteins (CSPs), and odorant binding proteins (OBPs), as well as detoxification-related genes of cytochrome P450 (P450), ATP-biding cassette (ABC), carboxyl/cholinesterase (CCE), UDP-glycosyltransferases (UGT), and glutathione-S-transferase (GST). In total, 30 GRs, 34 ORs, 36 IRs, 9 CSPs, and 17 OBPs were identified in *S. biformis* (Table 2). The gene family sizes of GRs, IRs, and ORs increased sequentially in *S. biformis*, *T. palmi*, and *F. occidentali*, while they remained largely unchanged for OBPs and CSPs (Table 2). This suggests that the gene family sizes of chemosensory-related genes, particularly GRs, IRs, and ORs, were positively related to the host range of these three Thripidae species. Phylogenetic analysis indicated that GRs expanded in *F. occidentali* and *T. palmi*, particularly in the sublineages of putative bitter and carbon dioxide receptor genes (Fig. 6a). Multiple ORs sublineages also expanded in *F. occidentali* and *T. palmi* (Supplementary Fig. 4). IRs expanded in *F. occidentali* primarily in the clade of divergent proteins (Supplementary Fig. 5). These sublineage expansions might be involved in polyphagous hosts of *F. occidentali* and *T. palmi*. Nevertheless, the phylogenetic tree of IRs and ORs revealed two *S. biformis*-specific sublineage expansions, which might be related to species-specific identification, like the perception of rice and other Poaceae plants. (Supplementary Figs. 4 and 5).

**Table 2:** Chemosensory-related and detoxification-related genes identified in three Thripidae species.

	Chemosensory-related					Detoxification-related				
	GR	OR	IR	CSP	OBP	P450	ABC	CCE	UGT	GST
*S. biformis*	30	34	36	9	17	92	60	69	14	25
*T. palmi*	73	53	42	11	22	96	49	39	17	25
*F. occidentali*	102	84	167	7	11	112	45	50	30	26

It is important for insects to fight against the defensive chemicals of host plants and the insecticides applied by humans. In this study, 92 P450s, 60 ABCs, 69 CCEs, 14 UGTs, and 25 GSTs were identified in *S. biformis* genome (Table 2). *S. biformis* possessed the largest gene family of ABCs and CCEs (Table 2, Supplementary Fig. 6, Fig. 6b). Whereas *F. occidentali* and *T. palmi* possessed a slightly expanded gene family size of P450s and UGTs (Table 2, Supplementary Fig. 7). No variation was found in gene family size of GSTs across three species (Table 2). Phylogenetic analysis of CCEs showed a lineage-specific expansion of the dietary/detoxification class of CCEs genes in *S. biformis*, which might be crucial for the detoxification of host plant rice (Fig. 6b).

A further experiment was conducted to confirm the role of detoxification-related genes after insecticide treatment. We found that in the deltamethrin-treated *S. biformis* group, 56 genes were upregulated, and 59 genes were down-regulated compared to the control group (Supplementary Data 17). Among the up-regulated genes, we observed the upregulation of a detoxification-related gene, glutathione S-transferase. In the case of imidacloprid-treated *S. biformis*, a total of 76 genes were upregulated, while 52 genes were down-regulated compared to the control group (Supplementary Data 18). Notably, we observed the upregulation of two detoxification-related genes, UDP-glucuronosyltransferase and cytochrome P450 307a1, following imidacloprid treatment. To confirm the reliability of the transcriptome results, we conducted Quantitative real-time PCR (qPCR) experiment for 11 genes (Supplementary Fig. 8). The result suggests that our result was reliable.

## Discussion

This study aimed to generate a high-quality genome assembly of *S. biformis* by combining Illumina paired-end short-reads, PacBio long-read sequencing, and Hi–C technology. The contigN50, scaffold N50, and BUSCO results indicate the assembly’s good contiguity and accuracy, providing a valuable resource for future genetic research.

In the three Thripidae species examined, we observed a positive correlation between the chemosensory gene repertoire and host plant range, particularly in GRs, ORs, and IRs. This finding aligns with previous studies in Coleopterans^21^ and Lepidoptera^22,23^, which suggest gene duplication within these gene families to aid in adaptation to diverse host plants. Four Coleopteran species showed a correlation between the content of chemosensory gene families and host specificity, with host-specific insects exhibiting fewer of ORs, GRs, IRs, and OBPs genes than polyphagous species^21^. Suzuki et al. ^22^. analyzed the GRs repertoire in four butterflies, generalist *Vanessa cardui*, and three specialists, and found a larger GRs number the in the generalist *V. cardui*. The expansion of GRs genes was also found in polyphagous Noctuidae, *S. frugiperda* and *Hlicoverpa armiger*a compared with mono- and oligophagous *B. mori*, which may be an adaptation mechanism for these species to adapt to a wide range of host plants^23^.

However, this correlation was not evident for all species. For example, the identification of odorant and GRs in six *Papilio* butterfly species showed similar ORs and GRs in both generalist and specialist species^24^. Furthermore, the size and content of chemosensory gene families IRs, GRs, and ORs were relatively conserved among sixteen species of Anopheles, irrespective of their varying host ranges^25^. While the three Thripidae species in this study were representatives of three different genera, and there are many other species lines among them in the phylogenetic tree. Thus, additional species information is necessary to confirm the correlation between chemosensory gene family size and host ranges in Thripidae and Thysanoptera.

Detoxification-related genes, including CYP450s, UDP-glycosyltransferases, esterase genes, GSTs, and ATP-binding cassette transporters, play a crucial role in plant secondary metabolites and contribute to insecticide tolerance in insects. Previous studies on *F. occidentalis* demonstrated the upregulation of detoxification genes, including glutathione S-transferase S1, three UDP-glucuronosyltransferases, four CYP450s, and one member of the ABC transporter G family, following treatment with three insecticides^26^. In *T. palmi*, the spinetoram-resistant population exhibited differentially expressed genes associated with P450s, heat shock proteins, CCEs, and ABC transporters^17^. In this study, we identified three up-regulated detoxification-related genes, namely cytochrome P450 307a1, UDP-glucuronosyltransferase, and glutathione S-transferase 1 in *S. biformis* after insecticide treatment. The number of differentially expressed genes was relatively lower compared to previous studies. Paralysis or reduced mobility behavior in response to insecticides treatment was observed in *S. biformis* after insecticide treatment. Firstly, the *S. biformis* used in the experiment was a laboratory population, which may be more susceptible to pesticides. Secondly, studies conducted on *S. avenae* have suggested that the expression of tolerance-related genes might be influenced by the treatment duration, with some differential expression of detoxification-related genes observed only after 36 h of treatment^27^. It is possible that further prolonging the treatment time in *S. biformis* could reveal additional detoxification-related genes in the future.

Except for detoxification-related genes, other genes associated with insecticide tolerance after treatment were observed. Several heat shock proteins were upregulated after deltamethrin treatment (Supplementary Data 17). Previous studies have shown that in *Myzus persicae*, a heat shock protein, MpHsp70, was upregulated in response to the upregulation of H_2_O_2_ induced by lambda-cyhalothrin, another pyrethroid insecticide^28^. Therefore, the upregulation of these three heat shock genes in our study may also be related to oxidative stress triggered by deltamethrin treatment. Moreover, we discovered the upregulation of two cuticle proteins (Supplementary Data 17, 18), which could contribute to increased insecticide tolerance by reducing permeability. In *Culex pipiens pallens*, several cuticle proteins were found to be overexpressed, resulting in increased cuticle thickness in deltamethrin-resistant strains^29–31^. These findings suggest that, in response to deltamethrin and imidacloprid treatment, *S. biformis* exhibits not only the upregulation of detoxification-related genes but also a broader range of gene responses.

## Methods

### Sampling

*S. biformis* strain used in this study was initially collected from rice fields in Ningbo, China, in 2020 and subsequently reared in the laboratory for about 10 generations. The strain was reared with rice seedlings (Xiushui 134) under controlled conditions of 27 ± 0.5 °C, relative humidity >80%, and a 16-h photoperiod.

### Genome sequencing

Genomic DNA was extracted from approximately 2000 adult *S. biformis* using the Wizard^®^ Genomic DNA Purification Kit according to the manufacturer’s instructions. To construct the library, a total of ~3 μg genomic DNA was randomly sheared into fragments of ~20 kb. The SMRTbell library was constructed using the SMRTbell Express Template Preparation Kit 2.0. The prepared library was sequenced on the PacBio Sequel II Platform at Novogene (Beijing) Co., Ltd., and circular consensus (CCS) read were generated. A total amount of ~0.2 μg DNA was used for Illumina paired-end sequencing library preparation using NEB Next® Ultra™ DNA Library Prep Kit for Illumina (NEB, USA) following the manufacturer’s recommendations. Then the DNA libraries were sequenced on the Illumina platform NovaSeq 6000.

### Genome size estimation and draft genome assembly

To estimate the genome size and heterozygosity of *S. biformis*, we conducted a genome survey using Illumina short reads and gce v1.0.2^32^ with parameter ‘-k 17’. Subsequently, long reads generated by PacBio were used to perform de novo genome assembly of *S. biformis*. The draft genome was assembled using FALCON v1.8.1^33^ with parameters ‘length_cutoff = −1; genome_size = 300,000,000; seed_coverage = 80’. The preliminary draft genome was further polished by NextPolish v1.4.1^34^ to remove the potential base errors by running one round of long reads polishing and two rounds of short reads polishing. In this process, minimap2^35^ was used to align the long reads to the genome, bwa v0.7.17-r1188^36^ was used to map short reads, and SAMtools v1.16.1^37^ was used to do file formats conversion. HaploMerger2^38^ was used to reduce the heterozygosity by soft-masking the repeat content of the genome with the WinMasker command, followed by running hm.batchA1-3 to eliminate significant misjoins from the diploid assembly and hm.batchB1-5 to generate the haploid assembly. Finally, Purge Haplotigs v1.1.1^39^ was applied to obtain the haploid genome sequences with purge_haplotigs cov using parameters ‘-l 50 -m 65 -h 80‘, and purge_haplotigs purge with parameters ‘-t 60 -a 70’.

### Hi–C sequencing

The Hi–C (high-throughput chromatin conformation capture) technique was utilized to construct the chromosome-level genome assembly of *S. biformis*. Approximately 1000 male individuals were prepared for Hi-C library construction. The samples were mechanically disrupted by homogenizer and then incubated in 2% formaldehyde for cross-linking reaction. Following cross-linking, chromatin was digested with 400 U of MboI restriction enzyme (NEB), Biotin labeling was performed to prepare the Hi–C samples, followed by DNA ligation with T4 DBA ligase (NEB), reverse cross-linking, DNA purification, shearing, and DNA ends repair. Biotin-labeled Hi–C samples were sequenced on the HiSeq-2500 platform to obtain 150 bp paired-end reads.

### Hi–C assembly

We used the ALLHic v0.9.8^40^ to perform chromosome construction with default parameters based on the Hi–C reads. We employed Juicebox Assembly Tools (JABT) v1.11.08^41^ to manually visualize and correct assembly errors. The final chromosome-level genome assembly was obtained by executing the run-asm-pipeline-post-review.sh script from 3D-DNA version 180922 (https://github.com/aidenlab/3d-dna).

### Transcriptome sequencing

For genome annotation, we sequenced the transcriptomes of 500 adult females and males separately. Total RNA was extracted using the Takara RNIzol Total RNA Isolation Kit, following the manufacturer’s instructions. The cDNA libraries were constructed by NEBNext^®^ Ultra™ RNA library prep kit for Illumina^®^ (NEB, USA) and then sequenced on the Illumina NovaSeq 6000. The cDNA library construction and sequencing were performed by Novogene Co., Ltd. (Beijing, China).

### Repetitive elements and noncoding RNA annotation

The repetitive and transposable elements were identified using both de novo and homology-based prediction methods. A de novo repeat library was constructed using RepeatModeler v2.0.1^42^ with parameter ‘-engine ncbi’. RepeatMasker v4.1.2^43^ was used to carry out homo-based repetitive elements prediction based on RepBase version 20181026 (http://www.girinst.org) with default parameters. Noncoding RNAs (ncRNA), such as miRNAs, snRNAs, and rRNAs were annotated using the Rfam database (http://rfam.xfam.org). Additionally, tRNAs were identified using tRNAscan-SE v2.0.9^44^ with default settings.

### Protein coding gene annotation

We utilized three lines of evidence to identify protein-coding genes, namely homo-based, RNA-based, and ab initio methods. For homo-based approaches, Gene Model Mapper (GeMoMa) v1.7.1^45^ was applied using *T. palmi*, *F. occidentalis*, *Drosophila melanogaster*, *Acyrthosiphon pisum*, and *Nilaparvata lugens* as references. For RNA-based methods, RNA transcriptome reads from females and males was assembled by TRINITY v2.11.0^46^ in genome-guided and no reference mode, respectively. To conduct genome-guided assembly, RNA-seq reads were mapped to the genome by Hisat2 v2.1.0^47^ with default parameters. We then used the PASA pipeline v2.4.1^48^ to align both genome-guided and de novo transcriptome transcripts to the genome with default parameters and obtain gene structures. Before ab initio prediction, repetitive elements from the whole genome were soft-masked. We utilized Augustus v3.3.3^49^ and SNAP v2006-07-28^50^, trained with 1365 genes selected from PASA results with CDS length > 1500 bp and exon number > 3. The gene set obtained from Augustus and SNAP was utilized by Maker version 3.01.03^51^ to generate an independent gene set. Furthermore, we utilized The transcriptome BAM files generated by Hisat2 were used by Braker v2.1.5^52^ to get another gene set with ‘--softmasking’. Finally, we integrated the independent gene sets above with EVidenceModeler v1.1.1^53^, using parameters ‘--segmentSize 1000000 --overlapSize 10000’, and weights ‘ab initio, 1; protein, 5; transcript, 10’. In the final gene set, we only retained genes with either transcript or homology evidence.

The gene functions of protein-coding genes were predicted by searching against the UniProtKB/Swiss-Prot^54^, UniProtKB/TrEMBL^55^, and nonredundant protein sequence (NR)^56^ databases using diamond v2.0.15^57^ using parameters ‘-sensitive; -evalue 1e-5; -max-target-seqs 20’. Additionally, interproscan v5.56-89.0^58^ was used to search against six databases: CDD, Gene3D, Panther, Pfam, SMART, and SUPERFAMILY. We also obtained the gene ontology (GO) and Kyoto Encyclopedia of Genes and Genomes (KEGG) annotations by aligning them to the EGGNOG (http://eggnog.embl.de) database using emapper v2.1.7^59^ with default parameters.

### Orthology, synteny, and phylogenetic reconstruction

Orthologues and orthogroups of 19 insect species were identified using OrthoFinder v2.5.4^60^ using ‘-M msa’. The phylogenetic tree was reconstructed based on single-copy genes and designated *Ephemera Danica* as the outgroup. MAFFT v7.505^61^ was used to align the homologous regions of the 1:1:1 orthologous gene using L-INS-I strategy. FastTree version 2.1.11^62^ was applied to infer an approximately-maximum-likelihood phylogenetic tree with JTT (Jones–Taylor–Thorton) amino acid evolution model and a single rate for each site (CAT). This process was a workflow in OrthoFinder. Additionally, we inferred the divergence time of these species using MCMCtree in paml version 4.9i^63^. Nucleotides of the 1:1:1 orthologous gene were multi-aligned by muscle v3.8.31^64^. For calibration of divergence time, six standard time points from TimeTree database (http://timetree.org) were used, including *A. pisum*–*T. palmi*, 206.1–404.6 million years ago (Ma), *Bombyx mori*–*Anopheles stephensi*, 223.8–344.7 Ma, *B. mori*–*Tribolium castaneum*, 280.9–361.6 Ma, *Apis mellifera*– *T. castaneum*, 312.9–389.7 Ma, *A. pisum*–*B. mori*, 330.4–481.7 Ma, *Ephemera danica*–*D. melanogaster*, 376.5–441.6 Ma. The phylogenetic tree was drawn by R package ggtree^65^.

In Thysanoptera, only *T. palmi* has a chromosome-level genome assembly. Therefore, we used *T. palmi* as the reference to investigate genome structure variation. We used BLASTP^66^ with parameters ‘-evalue 1e-5 -outfmt 6 -num alignments 10’ to compare the protein sequences of *S. biformis* and *T. palmi*. We then employed MCScanX^67^ to analyze the collinearity between these two species using gff file and blast results as inputs. The collinearity diagram was visualized by Circos v0.69-8^68^.

### Gene family expansion and contraction analyses

Computational Analysis of gene Family Evolution (CAFE) v4.2.1^69^ was used to characterize orthologous gene family contractions and expansions. To avoid non-informative parameter estimates, the clade_and_size_filter.py script supplied by CAFE was applied to filter out gene families with more than 100 gene copies in one or more species. A NEWICK format phylogenetic tree with divergence time as branch length was used as the input tree. The genetic birth and death rate of lambda value was estimated using ‘lambda -s’. GO and KEGG enrichment analyses were conducted using the online platform Omicshare (https://www.omicshare.com/tools). GO terms and KEGG pathways with *q-*value false discovery rate < 0.05 was considered significant.

### Gene family annotation of chemosensory-related and detoxification-related genes

To improve annotation accuracy and detect previously unidentified gene families, we re-annotated ten gene families associated with host perception (ORs, GRs, IRs, OBPs, and CSPs) and detoxification (P450s, ABCs, CCEs, UGTs, and GSTs). Gene family Hidden Markov models (HMMs) were downloaded from Pfam 35.0 (November 2021, 19,632 entries)^70^, and orthologs from model species *D. melanogaster*, as well as closely related species *T. palmi*, and *F. occidentalis*, were used for gene identification. We used the BITACORA^71^ pipeline to identify gene families and employed HMMER v3.3.1 and BLAST v2.11.0 for analysis. Protein sequences detected with conserved pfam domains and BLAST *E*-value < 1e−5 were considered as potential hits. We aligned genes using MUSCLE v3.8.31 and inferred the maximum likelihood phylogenetic tree for each gene family using IQ-TREE v1.6.12^72^ with 1000 bootstrap replicates.

### Transcriptome analysis of insecticide response genes

Two commonly used insecticides to control *S. biformis*, Imidacloprid (97% purity, Jiangsu Juhe Biological Agriculture Co., Ltd., Jiangsu, China) and Deltamethrin (98% purity, Nanjing Hongtaiyang Co., Ltd., Jiangsu, China), were employed in this study. Acetone (purity ≥ 99.8%, Xilong Scientific Co., Ltd., Guangdong, China) was utilized to dissolve the insecticide powder into appropriate concentrations. Previous studies have suggested that sublethal concentration (LC_10_) insecticide treatments can trigger the expression of detoxification-associated genes and insecticide tolerance^73^. Consequently, we conducted a pre-test to determine the LC_10_ concentration. The bioassays followed the procedures of Gao et al.^73^. Briefly, imidacloprid and deltamethrin powder were dissolved in stock solutions of 2000 ml/L using acetone and then serially diluted into different concentrations. For each insecticide, 100 µl of each dilution was used to coat the inner wall of 5 ml glass vials (Sangon Biotech Co., Ltd., Shanghai, China) by rolling for 5 min and then placed on a 3D rotating mixer (Hangzhou Miu Instruments Co., Ltd., Zhejiang, China) for 1 h to allow complete acetone evaporation. To prevent desiccation-induced mortality of *S. biformis*, 1 µl of double-distilled water was added to a piece of filter paper and placed in each vial, which was then sealed with parafilm. For the pre-test, 10–15 female *S. biformis* were introduced into each vial, and two replicates were prepared for each treatment. For the control group, the glass vials were only coated with 100 µl of acetone. Morality was recorded 8 h later. We ultimately determined concentrations of 2E−06 mg/L and 2E−05 mg/L for imidacloprid and deltamethrin, respectively (Supplementary Fig. 9). The same method as above was used to collect the insecticide-treated thrips for transcriptome analysis. Fifty females *S. biformis* were placed into each glass vial, and each treatment had three replicates. After 8 h of treatment, the samples were collected in TRIzol reagent. The transcriptome sequences were obtained using the same procedures described in the transcriptome sequencing section above.

To perform differentially expressed gene analysis, RNA-seq data were aligned to the reference genome using HISAT2 v2.1.0^47^ with default parameters. Subsequently, featureCounts v2.02^74^ was employed to determine the raw read count of each gene with parameters ‘-p -B -C -t exon -g gene_id’. The identification of differentially expressed genes between insecticide-treated and control groups was performed using the R package^75^ EdgeR v3.32.1^76^. The genes with normalized counts per million (CPM) value > 1 in at least two biological replicates were retained. The tagwise method was applied to estimate the dispersion, and a generalized log-linear model was used to fit the one-factor analysis. In accordance with a previous study^26^, genes exhibiting fold changes of <−1.5 or >1.5 were considered differentially expressed. Spearman’s rank correlation analysis was used to assess the correlation coefficients among the three replicates (Supplementary Data 19).

### Quantitative real-time PCR

The RNA used for transcriptome sequencing was utilized. A total of 1 μg RNA was reverse transcribed using the PrimeScript first-strand cDNA synthesis kit (TaKaRa, catalog No. 6110 A), following the manufacturer’s instructions. Gene primers were designed using Primer Premier version 5.0^77^. The internal reference gene used was the *actin* gene of *S. biformis* (see Supplementary Table 1 for primers list). qRT-PCR was conducted using SYBR Premix ExTaq Kit (TaKaRa) following the manufacturer’s instructions. The relative quantitative method 2^−ΔΔCt^ was utilized for calculating the relative expression variations^78^.

### Statistics and reproducibility

The GraphPad Prism 8 software^79^ was used to display the statistical analysis of qRT-PCR results (Supplementary Fig. 8). The bar graph was generated by representing the mean ± standard error. The statistical significance of differences between the control group and the insecticide-treated group was determined using the Student’s *t*-test (*P < 0.05; **P < 0.01; ***P < 0.001), each group consisted of three biological replicates.

### Reporting summary

Further information on research design is available in the Nature Portfolio Reporting Summary linked to this article.

### Supplementary information

Supplementary Information

Description of Additional Supplementary Files

Supplementary Data 1-19

nr reporting summary

## Data Availability

The raw sequences of PacBio, Illumina, and Hi–C data for genome assembly and RNA-seq data of insecticide treatment have been deposited in the Sequence Read Archive (SRR25338378, SRR25338348, SRR25338347, SRR24847376, SRR24847375, SRR24847374, SRR24847373, SRR24847372, SRR24847371, SRR24847379, SRR24847378, and SRR24847377) at NCBI under project PRJNA901696. This Whole Genome Shotgun project has been deposited at DDBJ/ENA/GenBank under the accession JAPMNG000000000. The version described in this paper is version JAPMNG010000000. The genome annotation file and manually annotated gene family sequences are deposited in figshare (10.6084/m9.figshare.23708619.v1). Source data underlying Fig. 3 are presented in Supplementary Data 10.
