# Supplementary material for: A chromosome-level genome assembly of Stenchaetothrips biformis and comparative genomic analysis highlights distinct host adaptations among thrips
Source: Commun Biol. 2023 Aug 4;6:813. doi: 10.1038/s42003-023-05187-1 (PMC10403496; doi:10.1038/s42003-023-05187-1)
Supplement: Supplementary file 1 — Supplementary Information [file 42003_2023_5187_MOESM1_ESM.pdf]

Supplementary Information

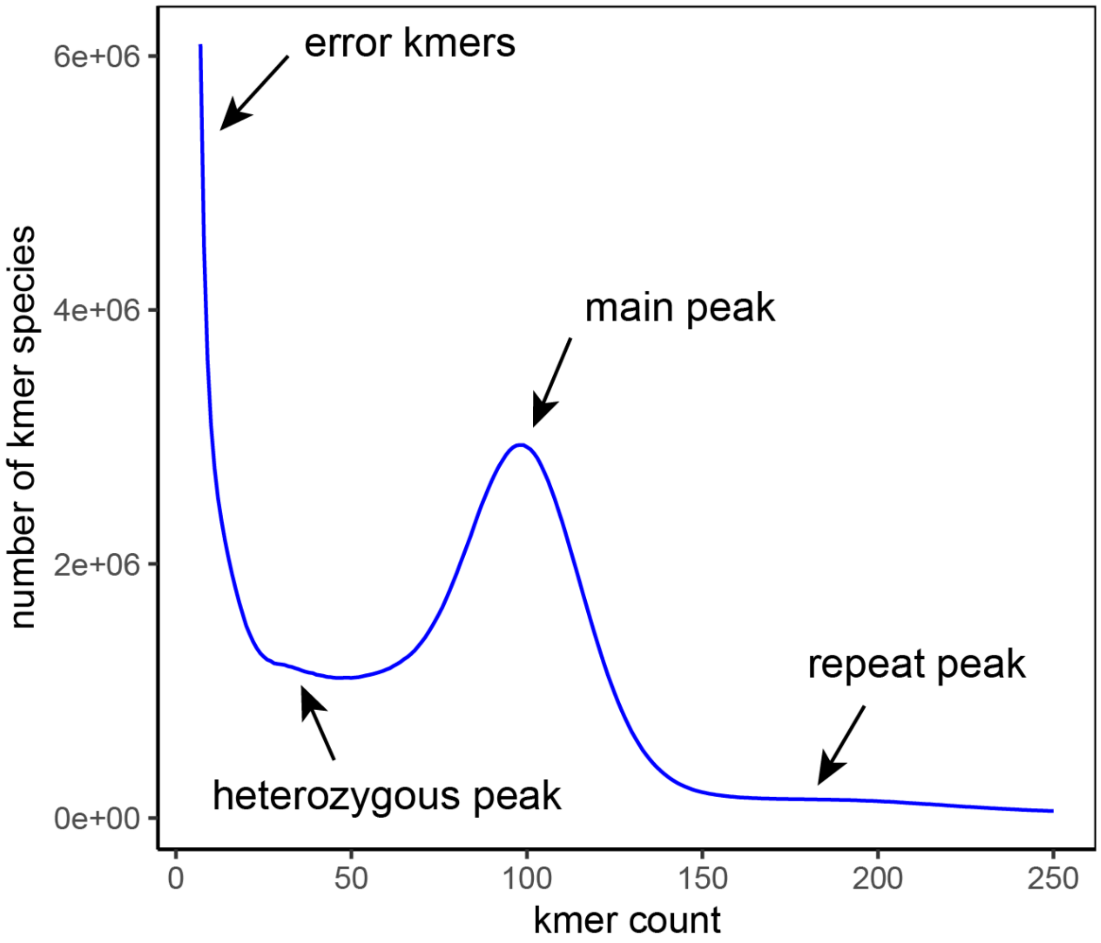

Supplementary Figure 1. K-mer distribution based on Illumina short paired-end reads to estimate the genome size and heterozygosity by gce-v1.0.2 with kmer set as 17.

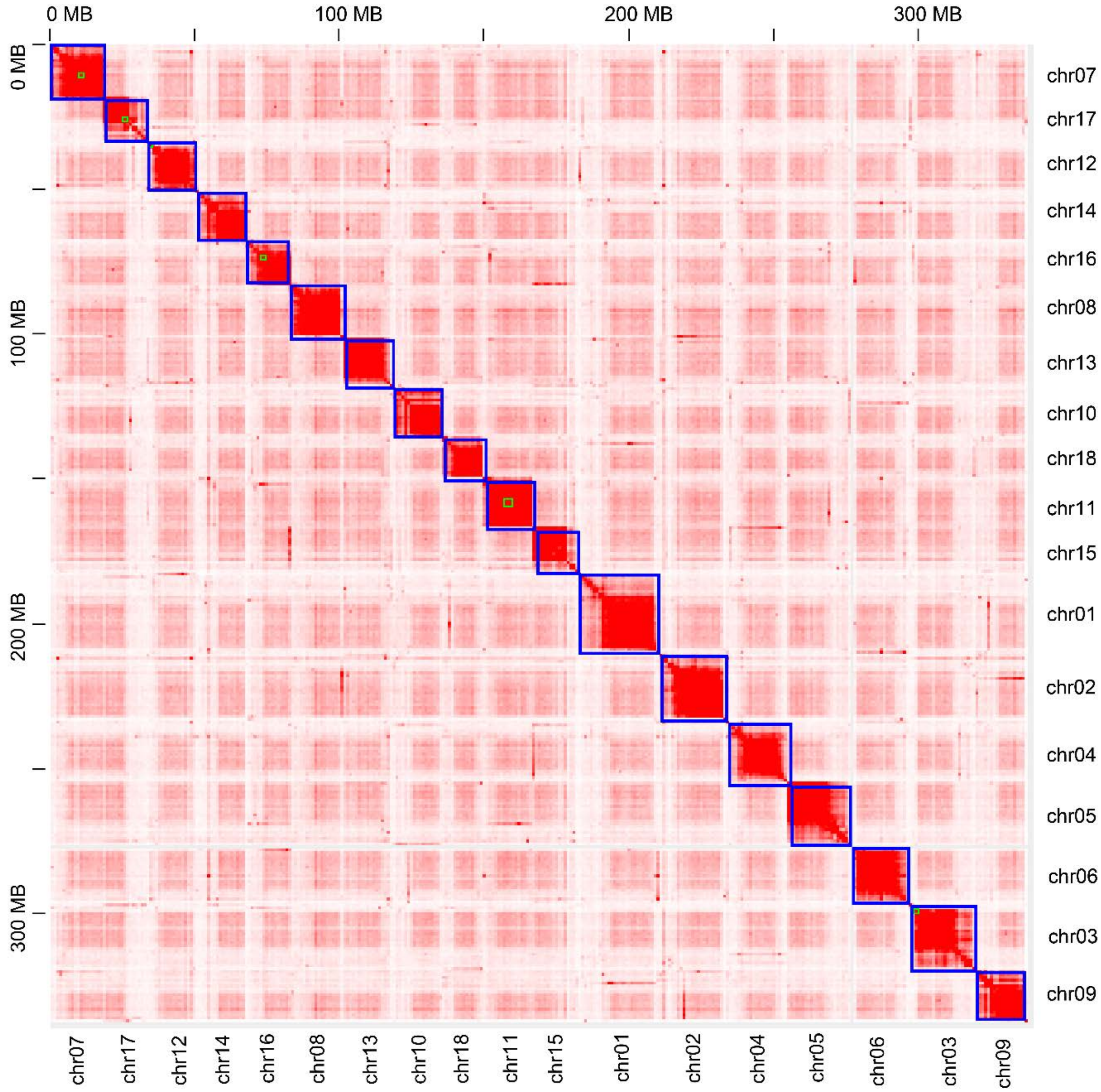

Supplementary Figure 2. The heatmap shows the frequency of all-by-all Hi-C contacts along the genome assembly of *S. biformis*. The chromosomes were renamed in sequence of chromosome length.

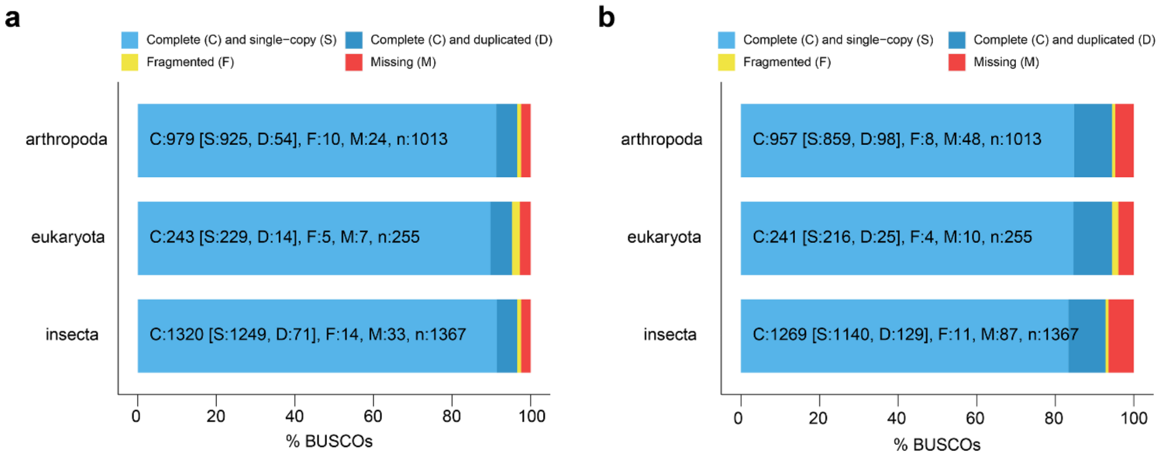

Supplementary Figure 3. **The completeness assessment of *S. biformis* genome assembly and gene annotation using BUSCO. (a)** The assembled genome was assessed using the Eukaryota (n=243), Arthropoda (n=979), and Insecta (n=1,320) gene sets. **(b)** The completeness of protein-coding genes was assessed using the Eukaryota (n=241), Arthropoda (n=957), and Insecta (n=1,269) gene sets.

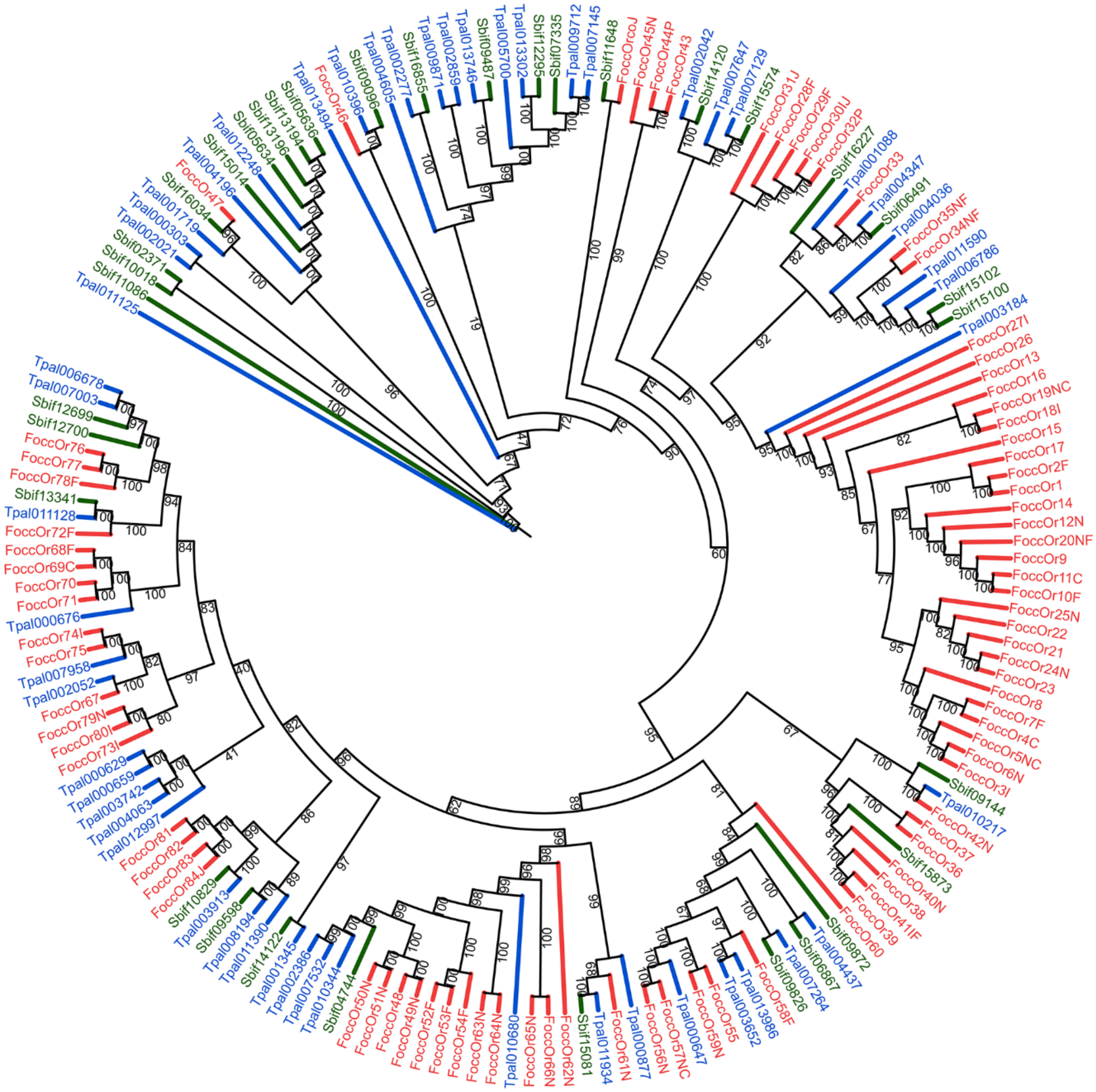

Supplementary Figure 4. Maximum-likelihood phylogenetic tree of ORs genes. ORs phylogenetic relationships of *S. biformis* were analyzed with *F. occidentali* and *T.palmi* using IQ-TREE with 1000 bootstrap replicates.

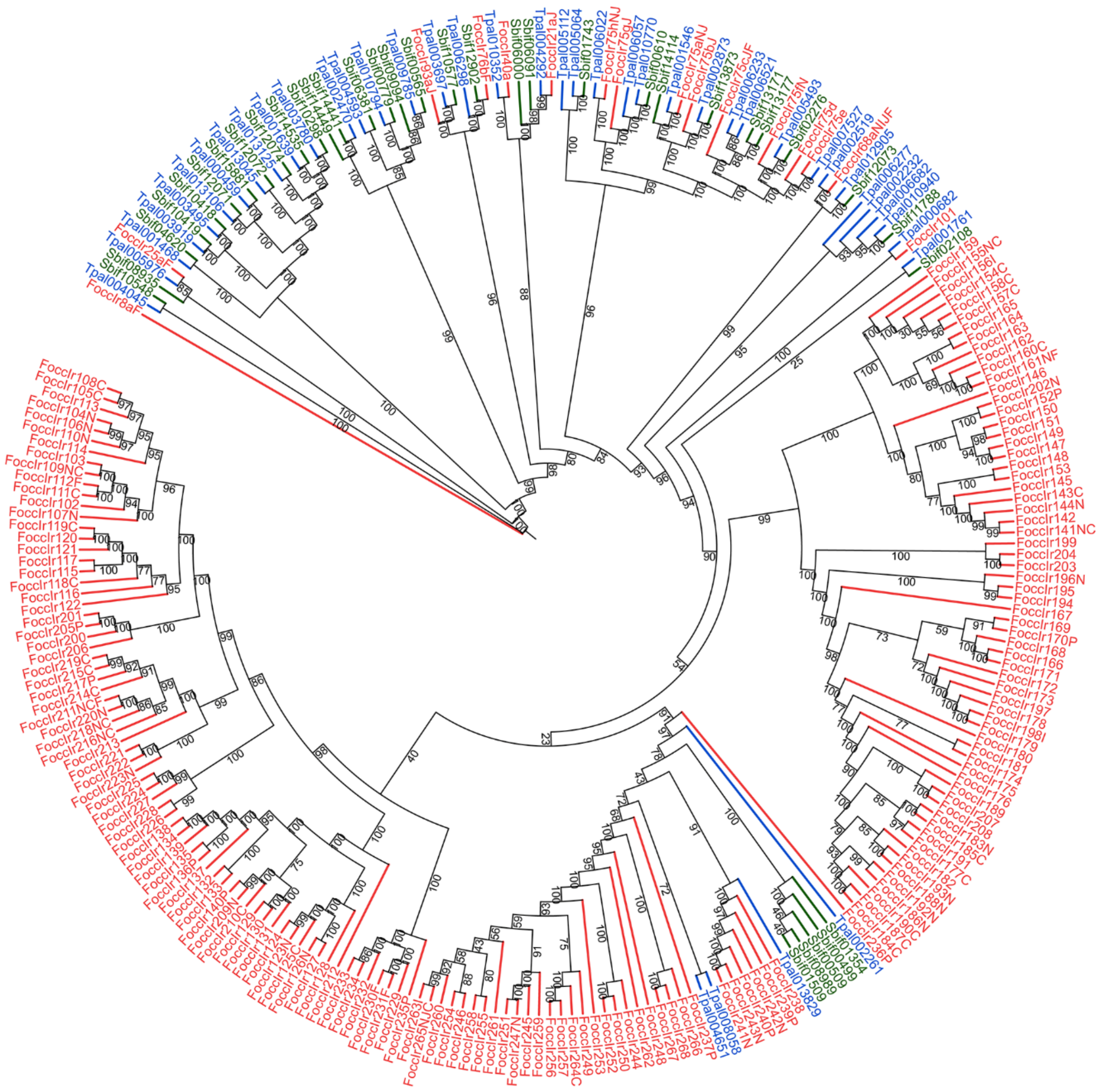

Supplementary Figure 5. Maximum-likelihood phylogenetic tree of IRs genes. IRs phylogenetic relationships of *S. biformis* were analyzed with *F. occidentali* and *T.palmi* using IQ-TREE with 1000 bootstrap replicates.

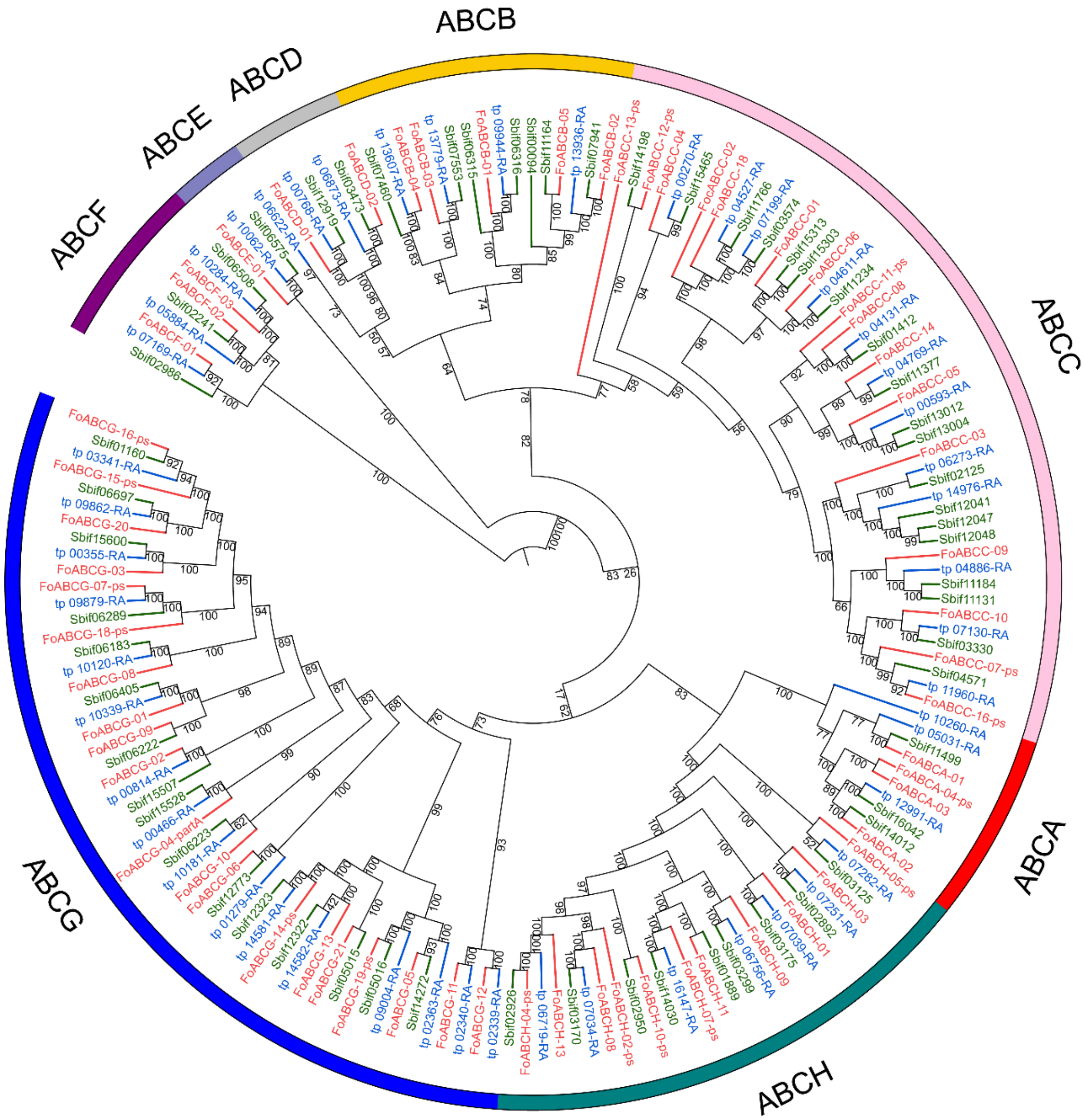

Supplementary Figure 6. **Maximum-likelihood phylogenetic tree of ABCs genes.** ABCs phylogenetic relationships of *S. biformis* were analyzed with *F. occidentali* and *T.palmi* using IQ-TREE with 1000 boostrap replicates.

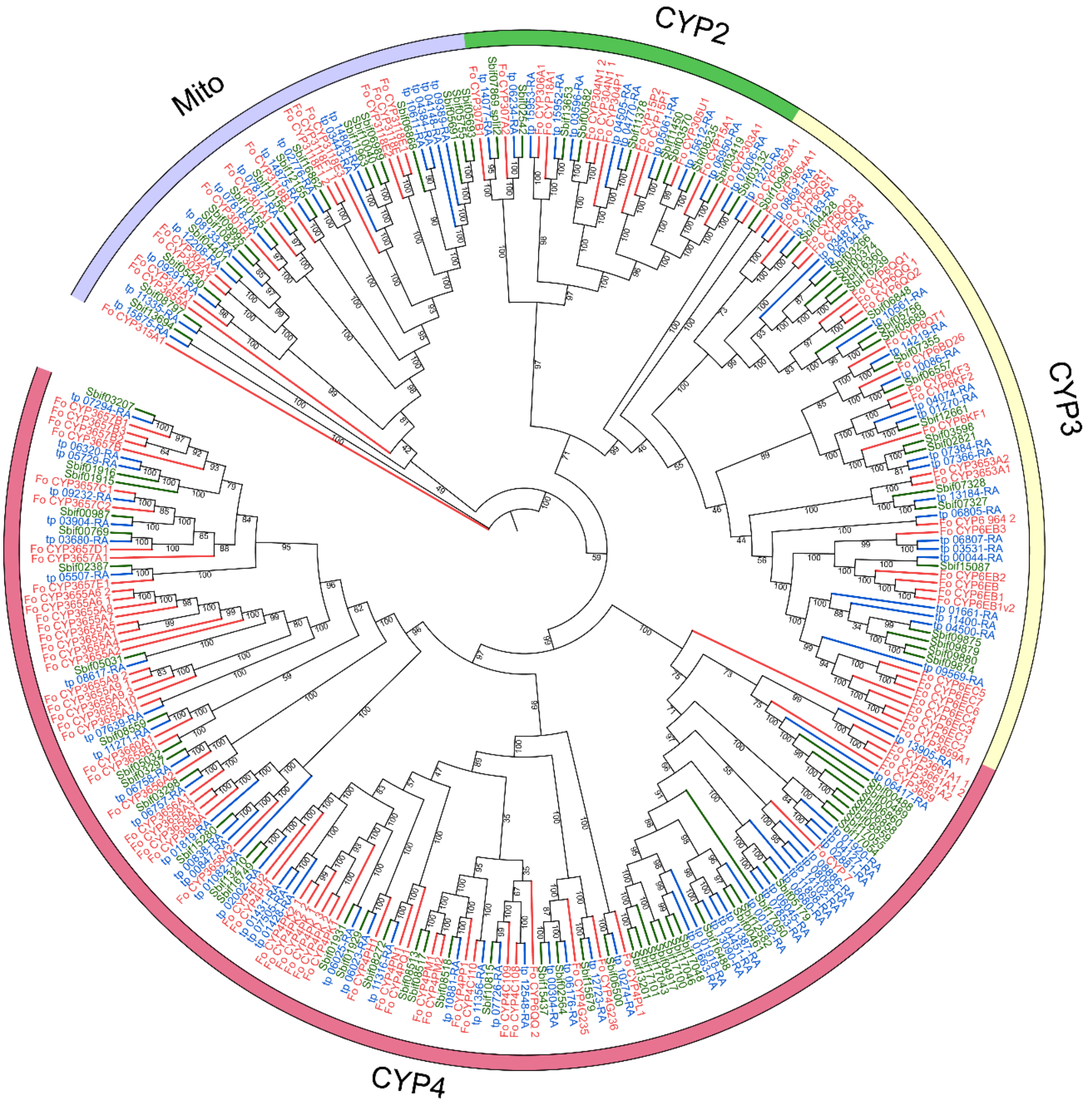

Supplementary Figure 7. Maximum-likelihood phylogenetic tree of P450 genes. P450s phylogenetic relationships of *S. biformis* were analyzed with *F. occidentali* and *T.palmi* using IQ-TREE with 1000 bootstrap replicates.

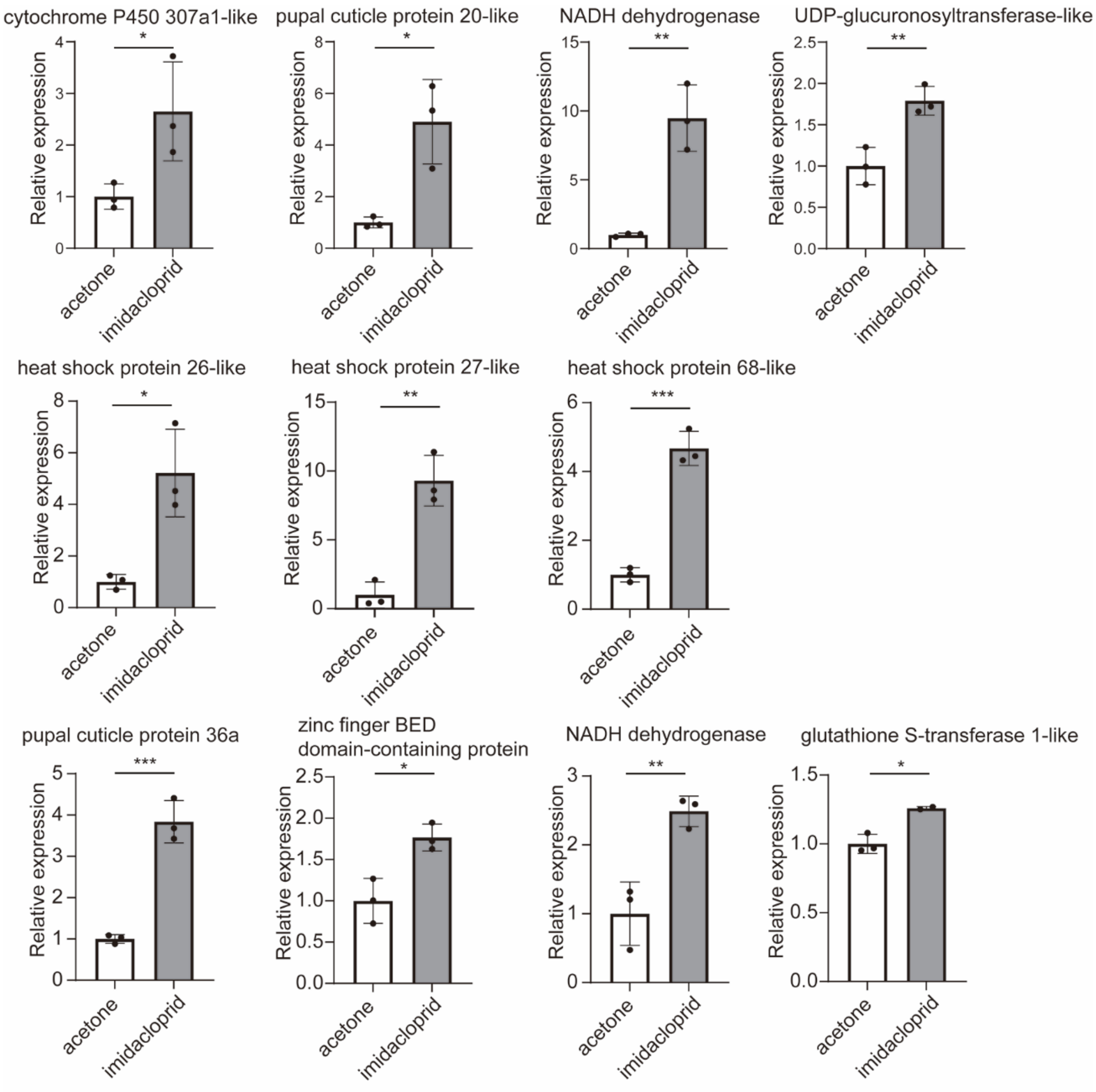

Supplementary Figure 8. Real-time Quantitative PCR results of 11 genes. Asterisks indicate significant difference between the control group and the insecticide treatment, determined using Student's *t*-test. *, $P < 0.05$; **, $P < 0.01$; ***, $P < 0.001$. The error bars represent the mean ± standard error, with n=3 biologically independent samples.

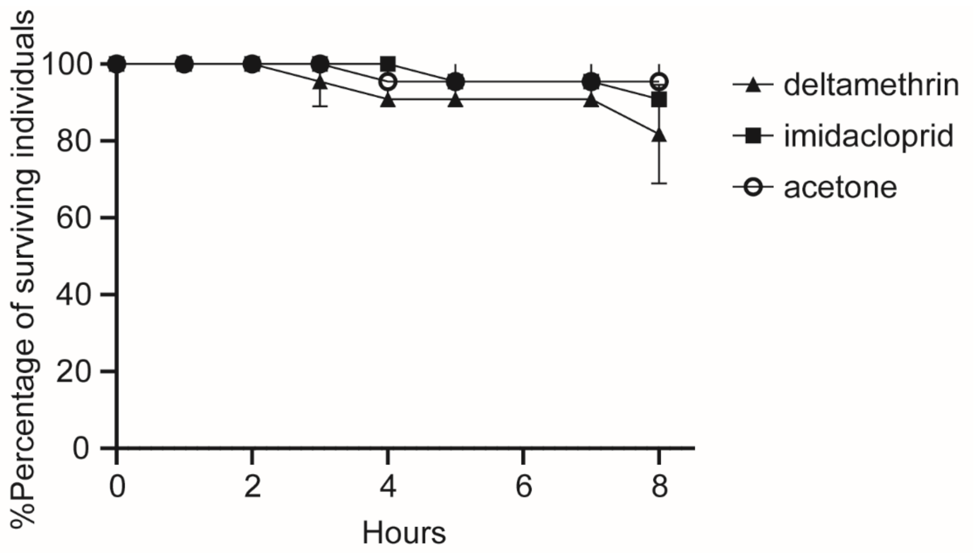

Supplementary Figure 9. Surviving rate of *S. biformis* following insecticide treatment. The insecticide concentrations were 2E-06mg/L and 2E-05 mg/L for imidacloprid and deltamethrin, respectively. Paralysis or reduced mobility behavior, followed by eventual death, was observed after both insecticide treatments. Although one individual in the control group also died, but no poisoning phenomenon was observed. The error bars represent the mean ± standard error.

Supplementary Table 1. Primers used for qPCR in this study.

Genes	Forward primers (5’-3’)	Reverse primers (5’-3’)
cytochrome P450 307a1	CCCATCATCGGCAACCTG	TCGGACCAATCGCACAGC
pupal cuticle protein 20	GACCAACGTCAACAACGG	GTGTACGAGAAGGAGCCC
NADH dehydrogenase 1 beta subcomplex subunit 4	CGTGTCATTGAAGAGAAA	AAAGAAGAAGCCAGTTGT
UDP-glucuronosyltransferase	CGGGTTTCGTGGTTCTGT	CTCATCGGGTGCGGGTAG
heat shock protein 26	CATCACCTCCAGGCTCTC	TCTCACTGTCCTTTTCGT
heat shock protein 27	GAGCACGGCTTTGTGCGG	CGTTGGTCTCCTTGGGGG
heat shock protein 68	CACCACCCCCAGCTATGT	TTCTTGTCCAGGCCGTAA
pupal cuticle protein 36a	CACAGGCCGGAGGTAGCA	CCGAAGCCGAAGGAAGAG
Zinc finger BED domain-containing protein 4	CGCCACCTCTGTAACCTC	CTCCCCCTAACCACTCCT
NADH dehydrogenase	TGAGCAAGAAAGAGCGAC	TTCATAATCATCTGGGGG
glutathione S-transferase 1-like	GTATCCCAAAGACCCGCA	AGAAGAGCCACCCCAACA
Actin	CCCATTGAGCACGGTATTGT	GGCTGGTGTGTTGAAGGTTT
