# Supplementary material for: A chromosome-level genome assembly of Stenchaetothrips biformis and comparative genomic analysis highlights distinct host adaptations among thrips
Source: Commun Biol. 2023 Aug 4;6:813. doi: 10.1038/s42003-023-05187-1 (PMC10403496; doi:10.1038/s42003-023-05187-1)
Supplement: Supplementary file 2 — Description of Additional Supplementary Files [file 42003_2023_5187_MOESM2_ESM.docx]

**Description of Additional Supplementary Files**

**File name:** Supplementary Data 1

**Description:** P Statistics of clean sequencing data used for S. biformis genome assembly.

**File name:** Supplementary Data 2

**Description:** Statistical results from the genome survey.

**File name:** Supplementary Data 3

**Description:** The statistics of contig-level assemblies.

**File name:** Supplementary Data 4

**Description:** Statistics of repetitive sequences by RepeatMasker.

**File name:** Supplementary Data 5

**Description:** The statistic of genome annotation.

**File name:** Supplementary Data 6

**Description:** The statistics for functional annotation.

**File name:** Supplementary Data 7

**Description:** Summary of ncRNA.

**File name:** Supplementary Data 8

**Description:** Species information used in this study.

**File name:** Supplementary Data 9

**Description:** Statistics of Gene family.

**File name:** Supplementary Data 10

**Description:** Statistics of genes for each species.

**File name:** Supplementary Data 11

**Description:** Significantly enriched GO terms of expansion gene families in S. biformis.

**File name:** Supplementary Data 12

**Description:** Significantly enriched KEGG pathways of expansion gene families in S. biformis.

**File name:** Supplementary Data 13

**Description:** Significantly enriched GO terms of expansion gene families in T. palmi

**File name:** Supplementary Data 14

**Description:** Significantly enriched KEGG pathways of expansion gene families in T. palmi.

**File name:** Supplementary Data 15

**Description:** Significantly enriched GO terms of expansion gene families in F. occidentali.

**File name:** Supplementary Data 16

**Description:** Significantly enriched KEGG pathways of expansion gene families in F. occidentali.

**File name:** Supplementary Data 17

**Description:** Differentially expressed genes in deltamethrin-treated and control group of S. biformis. Significance cutoff was logFC>1.5 and logFC<-1.5.

**File name:** Supplementary Data 18

**Description:** Differentially expressed genes in imidacloprid-treated and control group of S. biformis. Significance cutoff was logFC>1.5 and logFC<-1.5.

**File name:** Supplementary Data 19

**Description:** Pairwise Spearman's correlation between transcriptome biological replicates.
