# Supplementary material for: A chromosome-level genome assembly of Stenchaetothrips biformis and comparative genomic analysis highlights distinct host adaptations among thrips
Source: Commun Biol. 2023 Aug 4;6:813. doi: 10.1038/s42003-023-05187-1 (PMC10403496; doi:10.1038/s42003-023-05187-1)
Supplement: Supplementary file 4 — nr reporting summary [file 42003_2023_5187_MOESM4_ESM.pdf]

nature portfolio

Corresponding author(s): Chuan-Xi Zhang

Last updated by author(s): Jul 21, 2023

Reporting Summary

Nature Portfolio wishes to improve the reproducibility of the work that we publish. This form provides structure for consistency and transparency in reporting. For further information on Nature Portfolio policies, see our Editorial Policies and the Editorial Policy Checklist.

Statistics

For all statistical analyses, confirm that the following items are present in the figure legend, table legend, main text, or Methods section.

n/a	Confirmed	
☐	☒	The exact sample size (*n*) for each experimental group/condition, given as a discrete number and unit of measurement
☐	☒	A statement on whether measurements were taken from distinct samples or whether the same sample was measured repeatedly
☐	☒	The statistical test(s) used AND whether they are one- or two-sided<br>*Only common tests should be described solely by name; describe more complex techniques in the Methods section.*
☒	☐	A description of all covariates tested
☐	☒	A description of any assumptions or corrections, such as tests of normality and adjustment for multiple comparisons
☐	☒	A full description of the statistical parameters including central tendency (e.g. means) or other basic estimates (e.g. regression coefficient) AND variation (e.g. standard deviation) or associated estimates of uncertainty (e.g. confidence intervals)
☐	☒	For null hypothesis testing, the test statistic (e.g. *F*, *t*, *r*) with confidence intervals, effect sizes, degrees of freedom and *P* value noted<br>*Give P values as exact values whenever suitable.*
☒	☐	For Bayesian analysis, information on the choice of priors and Markov chain Monte Carlo settings
☒	☐	For hierarchical and complex designs, identification of the appropriate level for tests and full reporting of outcomes
☒	☐	Estimates of effect sizes (e.g. Cohen's *d*, Pearson's *r*), indicating how they were calculated

Our web collection on statistics for biologists contains articles on many of the points above.

Software and code

Policy information about availability of computer code

Data collection	All the software version and parameters used in this study were supplied in the Methods section of the manuscript.
Data analysis	All the software version and parameters used in this study were supplied in the Methods section of the manuscript.

For manuscripts utilizing custom algorithms or software that are central to the research but not yet described in published literature, software must be made available to editors and reviewers. We strongly encourage code deposition in a community repository (e.g. GitHub). See the Nature Portfolio guidelines for submitting code & software for further information.

Data

Policy information about availability of data

All manuscripts must include a data availability statement. This statement should provide the following information, where applicable:

- Accession codes, unique identifiers, or web links for publicly available datasets
- A description of any restrictions on data availability
- For clinical datasets or third party data, please ensure that the statement adheres to our policy

Genomes and associated data for Stenchaetothrips biformis are deposited at NCBI under project PRJNA901696.

Field-specific reporting

Please select the one below that is the best fit for your research. If you are not sure, read the appropriate sections before making your selection.

☒ Life sciences ☐ Behavioural & social sciences ☐ Ecological, evolutionary & environmental sciences

For a reference copy of the document with all sections, see nature.com/documents/nr-reporting-summary-flat.pdf

Life sciences study design

All studies must disclose on these points even when the disclosure is negative.

Sample size	We refered the sample size from previous study of the west flower thrips genome assembly to guarantee sufficient genomic DNA extraction.
Data exclusions	The sequencing raw data need quality control step to remove adapters and low-quality reads before used for genome assembly. The exclusion criteria were commonly used standards.
Replication	De novo genome assembly usually do not apply replication data. For insecticides treatment RNA-seq data, we prepared three biological samples.
Randomization	The samples were randomly collected for genome sequencing.
Blinding	No, this is not applicable for this study.

Reporting for specific materials, systems and methods

We require information from authors about some types of materials, experimental systems and methods used in many studies. Here, indicate whether each material, system or method listed is relevant to your study. If you are not sure if a list item applies to your research, read the appropriate section before selecting a response.

Materials & experimental systems

n/a	Involved in the study	
☒	☐	Antibodies
☒	☐	Eukaryotic cell lines
☒	☐	Palaeontology and archaeology
☐	☒	Animals and other organisms
☒	☐	Clinical data
☒	☐	Dual use research of concern

Methods

n/a	Involved in the study	
☒	☐	ChIP-seq
☒	☐	Flow cytometry
☒	☐	MRI-based neuroimaging

Animals and other research organisms

Policy information about studies involving animals; ARRIVE guidelines recommended for reporting animal research, and Sex and Gender in Research

Laboratory animals	This study did not involve in laboratory animals.
Wild animals	This study did not involve in wild animals.
Reporting on sex	We sequenced the mixed genomic DNA sample of female and male adults of rice thrips. Due to the sex determination way of this species was female diploid and males haploid.
Field-collected samples	S. biformi strain used in this study was initially collected from rice fields in Ningbo, China in 2020, and was reared in the laboratory for about 10 generations. The strain was reared with rice seedlings (Xiushui 134) at 27 ± 0.5 °C under a relative humidity of >80% and a 16-hr photoperiod.
Ethics oversight	The program of insect collection and experiment in the article has passed the ethical review of Animal and plant Ethics Committee of Ningbo University.

This checklist template is licensed under a Creative Commons Attribution 4.0 International License, which permits use, sharing, adaptation, distribution and reproduction in any medium or format, as long as you give appropriate credit to the original author(s) and the source, provide a link to the Creative Commons license, and indicate if changes were made. The images or other third party material in this article are included in the article's Creative Commons license, unless indicated otherwise in a credit line to the material. If material is not included in the article's Creative Commons license and your intended use is not permitted by statutory regulation or exceeds the permitted use, you will need to obtain permission directly from the copyright holder. To view a copy of this license, visit http://creativecommons.org/licenses/by/4.0/
